# Activating Phase-Transition Toughening in van der Waals Semiconductor GaTe

**DOI:** 10.1021/acs.nanolett.6c01935

**Published:** 2026-07-07

**Authors:** Ruihan Xu, Boxiang Gao, Danlei Zhao, Jingzhuo Zhou, Qi Zhu, Yupeng Ma, Binzhao Li, Yi Zhang, Juzheng Chen, Qian Zhang, Fanling Meng, Johnny C. Ho, Maolin Yu, Yang Lu

**Affiliations:** † Department of Mechanical Engineering, The University of Hong Kong, Hong Kong 999077, China; ‡ State Key Laboratory of Structural Analysis, Optimization and CAE Software for Industrial Equipment, Department of Engineering Mechanics, 665904 Dalian University of Technology, Dalian 116023, China; § Department of Materials Science and Engineering, 53025 City University of Hong Kong, Kowloon 999077, China; ∥ State Key Laboratory of High-performance Precision Manufacturing, Dalian University of Technology, Dalian 116023, People’s Republic of China; ⊥ Department of Mechanical Engineering, City University of Hong Kong, Kowloon 999077, China

**Keywords:** *in situ* mechanics, fracture toughness, phase transition, crack propagation

## Abstract

Inorganic semiconductors are essential for modern electronics, but their inherent brittleness restricts their applications in flexible and wearable devices. This issue is particularly acute in low-symmetry structures, where the lack of slip systems further suppresses plastic deformation. Here, we reveal an intrinsic toughening mechanism in monoclinic GaTe using *in situ* SEM microfracture experiments. In contrast to the catastrophic brittle cleavage along the interlayer direction, cross-layer crack propagation undergoes continuous deflection, generating a highly tortuous crack path that enhances the mean fracture toughness by ∼60%. Combining high-resolution imaging with atomic simulations, we identify stress-triggered monoclinic-to-trigonal phase transitions at deflection points, which effectively impede and redirect crack propagation. The toughening mechanism is further validated in a flexible GaTe photodetector, which retains an excellent photoresponse and mechanical durability over tens of thousands of bending cycles. These findings lay a solid foundation for nanodevice applications in which both mechanical robustness and functional stability are required.

## Graphical abstract

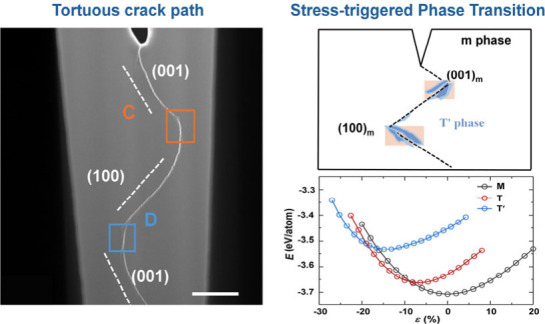

Inorganic semiconductors exhibit a rich spectrum of physical properties, making them ideal candidates for advanced photonic, electronic, and flexible-sensing architectures.^1,2^ However, the highly directional bonding nature gives rise to limited defect tolerance, rendering them mechanically vulnerable to stress concentration at flaws.^3,4^ For example, group IV elemental crystals and ceramic oxides typically experience brittle fractures along specific crystallographic planes, resulting in toughness that is 1–2 orders of magnitude lower than that of conventional metals.^5,6^ This fracture issue is particularly pronounced in two-dimensional (2D) van der Waals (vdW) semiconductors, where strong covalent intralayer bonding coexists with weak interlayer vdW interactions.^7^ The pronounced structural anisotropy makes these materials susceptible to interlayer cleavage, leading to catastrophic failure with minimal energy dissipation.^8^ Understanding these fracture mechanisms is, therefore, critical for enabling reliable device applications.^9^

Plastic deformation is a critical contributor to toughness as it enables the local dissipation of concentrated stresses. Recent studies reported that hexagonal layered metal chalcogenides, including InSe, GaSe, SnS_2_,^10–14^ and ZnS,^15^ can sustain substantial structural deformation under ambient conditions, which has been attributed to dislocation migrations,^15^ interlayer reconstruction, and cross-layer linking.^16–19^ Because of the scarcity of available slip systems, this deformability is not anticipated in low-symmetry crystals. However, stress-driven structural transitions have been observed in monoclinic Ag_2_S and GaTe.^20,21^ Given that strong structural anisotropy usually introduces competing crack propagation pathways, plastic deformation can locally alter stress fields, thereby influencing crack paths and promoting multiroute energy dissipation. Elucidating the interplay between plastic deformation and anisotropic fracture is essential for engineering the toughness of low-symmetry crystals. Monoclinic gallium telluride (m-GaTe) represents a significant class of 2D vdW crystals with low crystal symmetries and high in-plane anisotropy.^22^ This structural asymmetry is highly advantageous for developing advanced anisotropic nanoelectronics,^23^ such as polarization-sensitive photodetectors and nonvolatile memories.^24,25^ However, integrating these directional functionalities into next-generation flexible electronics strictly necessitates a fundamental understanding of the material’s fracture toughness, as structural anisotropy often introduces susceptible pathways for catastrophic failure.

Here, we systematically investigate the anisotropic fracture behaviors of low-symmetry monoclinic gallium telluride (m-GaTe) crystals using *in situ* measurements and atomic simulations. Micromechanical testing within scanning electron microscopy (SEM) reveals that m-GaTe favors brittle cleavage along the (201) planes, exhibiting minimal energy dissipation and a mean fracture toughness of 0.41 MPa m^0.5^. In contrast, cross-layer fracture promotes periodic crack deflection along the (100) and (001) planes, which effectively dissipates energy and enhances the mean fracture toughness to 0.67 MPa m^0.5^. High-resolution scanning transmission electron microscopy (STEM) characterizations uncover structural transitions from the low-symmetry monoclinic to the high-symmetry trigonal phase at crack deflection points. These transformations create nanoscale pinning sites that effectively arrest crack growth, as validated by our machine-learning-assisted molecular dynamics (MD) simulations. The anisotropic fracture behavior and toughening mechanisms enable m-GaTe photodetectors to withstand more than 10 000 bending cycles while maintaining their optoelectronic performance.

The ground state of GaTe preferentially adopts a low-symmetry monoclinic structure in space group *C*2/*m* (Figure 1a).^22^ Each layer is composed of Te–Ga–Ga–Te quadruple slabs built from corner-sharing GaTe_3_ tetrahedral motifs. Owing to the partial occupation of antibonding orbitals in polar Ga–Te bonds, one-third of the nonpolar Ga–Ga bonds rotate by ∼90° to mitigate antibonding penalties and enhance the chemical stability of m-GaTe.^20^ The lattice distortion leads to pronounced in-plane anisotropy, as confirmed by angle-resolved polarized Raman characterization in Figure 1b. At the A_g_ mode of 114 cm^–1^, the Raman intensity exhibits a periodic modulation with every 90°, reflecting inequivalent crystallographic directions with distinct bonding strengths. The lattice anisotropy provides a structural basis for the direction-dependent crack propagation in m-GaTe.

**Figure 1:**
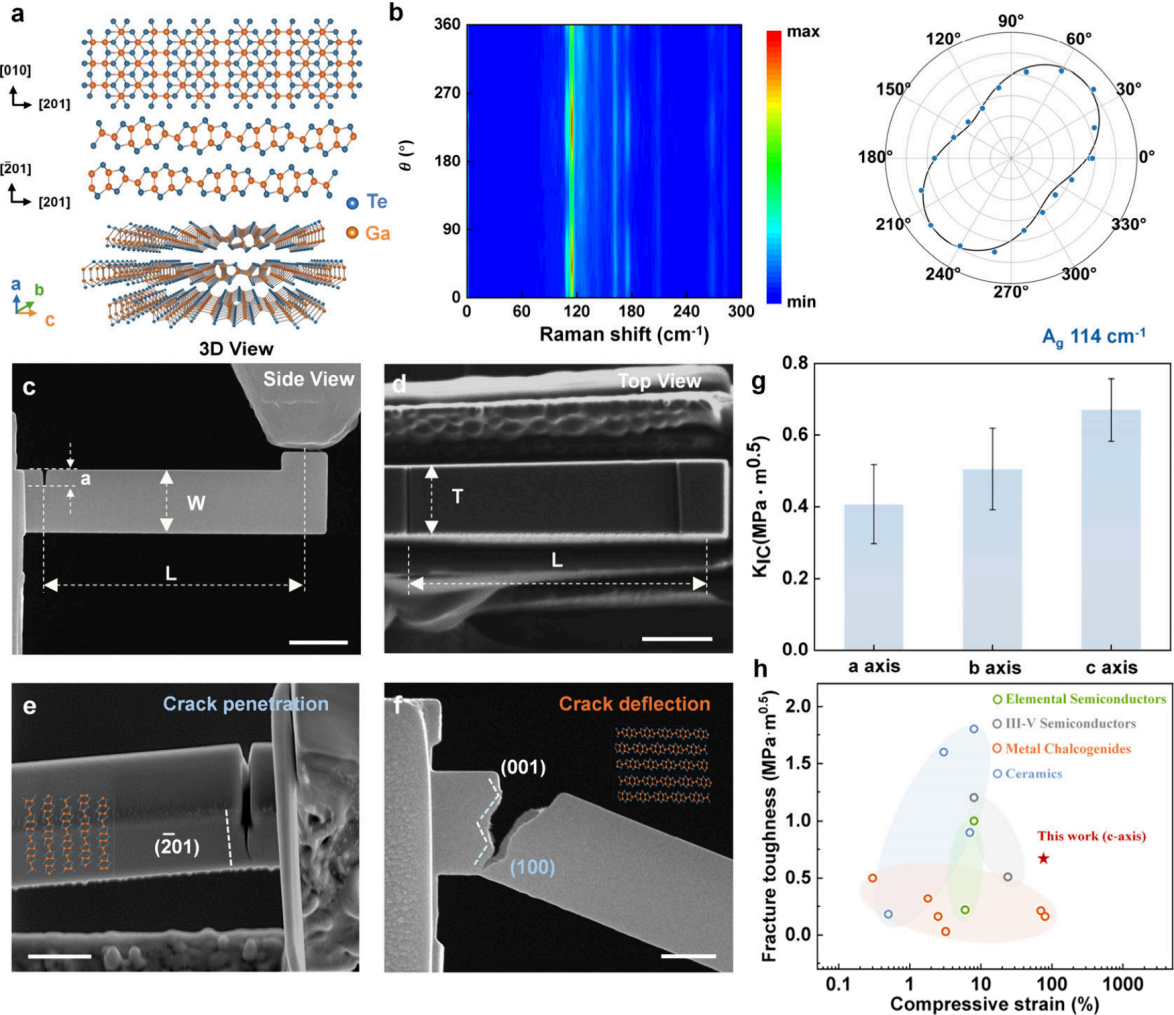
Anisotropic structures and fracture behaviors in m-GaTe crystals. (a) Atomic structure of m-GaTe, illustrating its intrinsically anisotropic bonding configuration. (b) Intensity map and polar plot of the angle-resolved polarized Raman spectroscopy characterization at the A_g_ mode of 114 cm^–1^. (c and d) Side and top views, respectively, of the *in situ* SEM microcantilever setup used for fracture toughness measurements. Scale bars, 1 μm. (e and f) Crack morphologies of m-GaTe showing interlayer cleavage along the (201) plane and cross-plane fractures along the (100) and (001) planes, respectively. Scale bars, 500 nm. (g) Measured critical stress intensity factor *K*_IC_ along different crystallographic directions. (h) Ashby plot showing the relationship between critical compressive strain and fracture toughness for inorganic semiconductors. Data from III–V compound semiconductors,^28–31^ ceramics,^5,32–36^ elemental semiconductors,^6,37,38^ and metal chalcogenides^9,10,12,13,20,39–49^ are included for comparison.

Using focused ion beam (FIB) milling, we fabricated microcantilevers to measure the anisotropic fracture behaviors of m-GaTe. A sharp notch was introduced at the root of each cantilever. Then, the single cantilever bending tests were performed inside the SEM, where a controlled displacement of the flat diamond indentor was applied to the free end at a constant rate of 4 nm/s (Figure 1c,d and Figure S1). Critical load *F* at fracture, together with cantilever geometry factor $f\left(\frac{a}{W}\right)$ , was used to calculate critical stress intensity factor *K*_IC_ according to the following linear elastic fracture mechanics equations.^26,27^

1 $$K_{\mathrm{IC}}=\frac{FL}{BW^{3/2}}f\left(\frac{a}{W}\right)$$

2 $$f\left(\frac{a}{W}\right)=1.46+24.36\left(\frac{a}{W}\right)-47.21{\left(\frac{a}{W}\right)}^{2}+75.18{\left(\frac{a}{W}\right)}^{3}$$

where *L*, *W*, and *B* are the length, width, and thickness of the cantilever, respectively, and *a* is the notch length (Table S1).

When the m-GaTe specimens are bent along the *a*-axis ([201] in Figure 1a), cracks maintain a linear propagation path and rapidly cut across the microcantilever (Figure 1e and Figure S1d). The resulting (201) fracture planes are predominantly smooth, reflecting a brittle cleavage governed by weak interlayer interactions. The measured fracture toughness is only 0.41 ± 0.11 MPa m^0.5^, closely matching the typical range of metal chalcogenides (0.03–0.50 MPa m^0.5^).^9^ Besides, bending along the *b*-axis also produces a straight crack path, attributed to the nearly isotropic bonding environment along the out-of-plane (201) plane (Figure S1e). Because fracture in this direction involves the rupture of covalent Ga–Te bonds rather than interlayer cleavage, the measured toughness increases modestly to 0.51 ± 0.11 MPa m^0.5^.

A pronounced toughening effect emerges when the specimens are bent along the *c*-axis. Although the initial notch is positioned on the (201) plane, the crack is redirected to the (100) plane and later turns toward the (001) plane under loading (Figure 1f), producing a tortuous cracking path. Crack deflection is consistently observed across multiple samples (Figure S1). The mechanism increases the effective fracture plane and energy dissipation, yielding an increased fracture toughness of 0.67 ± 0.09 MPa m^0.5^. The measured toughness of m-GaTe is summarized in Figure 1g, revealing the following clear directional dependence: *K*_IC‑c_ > *K*_IC‑b_ > *K*_IC‑a_. Furthermore, we benchmark the overall mechanical performance of m-GaTe against the conventional semiconductors. As shown in Figure 1h, m-GaTe not only exhibits a maximum toughness surpassing all reported transition-metal chalcogenides but also delivers plastic deformation capabilities well above those of typical inorganic semiconductors.^9,20^

To further investigate the fractographic features and atomic-scale microstructure, a double-cantilever beam (DCB) setup was prepared via FIB milling.^36,50^ The electron-transparent central region of this DCB setup was thinned to 100 ± 20 nm, with its horizontal and vertical axes aligned along the interlayer [201] and out-of-plane [201] directions, respectively. A sharp precrack (∼1 μm in length) was introduced at the sample center (Figure S2). To confirm the single-crystalline monoclinic structure and the absence of FIB-induced damage, the specimens of microfracture tests were characterized, as shown in Figure S3. A wedge-shaped diamond indenter (half-angle of ∼55°) was placed on the top of the DCB and then advanced downward at a constant speed of ∼4 nm/s (Figure 2a).

**Figure 2:**
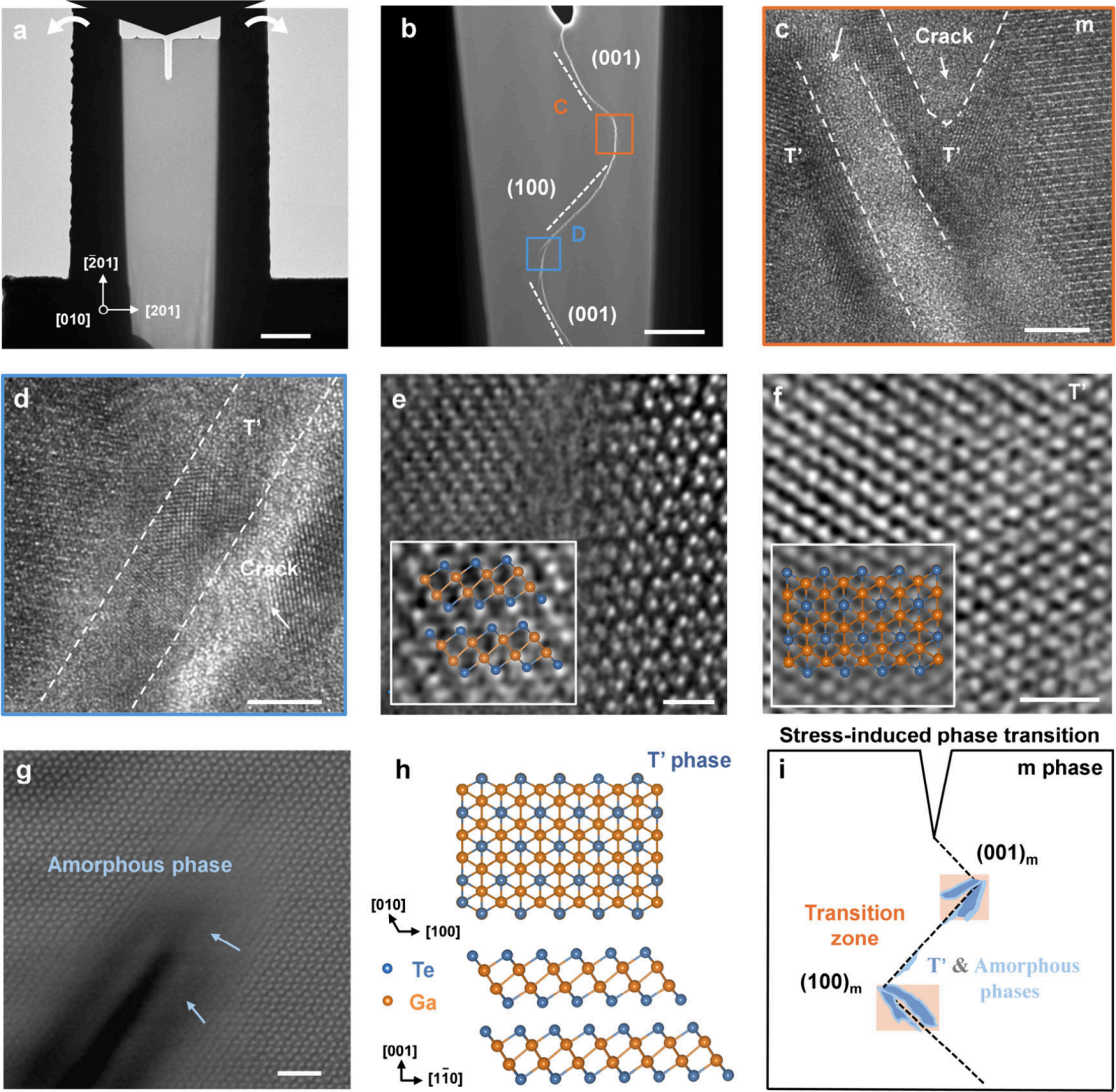
Crack arrest and deflection in monoclinic GaTe. (a) Experimental setup for DCB fracture testing. Scale bar, 1 μm. (b) Cross-sectional view of crack growth along the out-of-plane (010) direction. Scale bar, 700 nm. (c and d) HRTEM images of the deflection regions along the crack path. Scale bars 6 and 4.2 nm in panels c and d, respectively. (e and f) High-resolution STEM-HAADF images of the transition region, captured along the (110) and (001) planes, respectively, of the T′ phase. Scale bars, 1 nm. (g) Crystalline-to-amorphous phase transitions at the crack tip. Scale bar, 2 nm. (h) Atomic structure of the newly identified T′ phase. (i) Illustration of intrinsic toughening driven by the monoclinic-to-trigonal and crystalline-to-amorphous phase transitions.

In this setup, the vertical compression is transformed into horizontal tensile stress, initiating the crack propagation from the [201] notch. As observed in Figure 2b, the crack proceeds initially along the (001) plane, deflects onto the (100) plane upon blockage, and then redirects back to the (001) plane under continued loading. The resulting periodic crack deflections, characterized by a wavelength of approximately 1 μm, qualitatively match the fracture patterns observed in single-cantilever bending tests (Figure 1f). More interestingly, we find that the primary crack stalls precisely at these deflection points. In contrast, several secondary cracks emerge and propagate. This behavior implies that the energy required for crack growth along the original path far exceeds that required to create new fracture pathways.

To elucidate the atomic mechanisms governing crack arrest and deflection, we conducted high-resolution transmission electron microscopy (HRTEM) characterization of the deflection regions, which are marked by the orange and blue boxes in Figure 2b. As shown in Figure 2c and Figure S4a, the crack advances through the m-GaTe lattice, with transition regions appearing between the crack path and the surrounding matrix. A similar phenomenon is observed in another deflection region shown in Figure 2d and Figure S4b. Then, we utilized high-angle annular dark-field scanning transmission electron microscopy (HAADF-STEM) to probe the atomic structure of the transition region, as highlighted by the white box in Figure 2e. Characterization along the interlayer direction (the [010] zone axis) reveals a pronounced transition from the monoclinic to the trigonal phases (Figure 2e), while the in-plane projection (along the [001] zone axis) shows well-defined triangular lattices that are entirely distinct from those of the m-GaTe structures (Figure 2f).

Guided by the atomic-resolution images, we propose a structural model for the trigonal phase, as illustrated in Figure 2h. The newly identified phase crystallizes in the *R* 3 *m* space group, which is denoted as the T′ phase to distinguish it from the previously reported trigonal structure.^18,51^ The measured lattice constant and interlayer distance of the T′ phase are 4.12 and 7.40 Å, respectively, in excellent agreement with the first-principles calculations (4.10 and 7.53 Å, respectively, in Figure S4c). Besides, cracking-induced amorphization was observed near the crack path, occurring with and without adjacent T′ phases (Figure 2g and Figure S4d). As shown in Figure 2i, these findings indicate pronounced stress concentration at the crack tip, while the monoclinic-to-trigonal and crystalline-to-amorphous phase transitions act as effective pathways for stress dissipation, thereby promoting improved fracture toughness.^11,16^

To investigate the nucleation and toughening mechanisms associated with the phase transition, we first used the finite element method (FEM) to characterize the strain field distribution during crack propagation. In anisotropic materials, directional stiffness mismatch induces tension-shear coupling and incompatible deformation, generating compressive stresses at the crack tip even under pure mode I loading.^52^ As shown in Figure S5, localized compressive strains reaching ∼7.0% emerge at the deflection sites, consistent with previous reports.^8,53^ Compounded by the intrinsic crack tip singularity, the actual near tip strain is expected to be more intensely amplified than the simulation results.^54^ We therefore performed the first-principles calculations to probe the structural response of GaTe crystals to localized compressive strain. The relative energies of three competing phases, including the monoclinic (m), conventional trigonal (T), and newly identified T′ phases, were examined under in-plane compression. The T phase was considered an intermediate state between the m and T′ phases, as reported previously.^51,55^ The equilibrium lattice parameters of the m phase were used as the reference for the zero-strain condition. As depicted in Figure 3a, the energy evolution of these phases reveals two successive phase transitions with an increase in compressive strain. The m phase, initially the most stable, becomes higher in energy than the T phase after a critical strain of −8.1%. Upon further compression to −18.8%, the T phase is superseded by the T′ phase. These results indicate that the accumulated strain energy can be consumed by the nucleation and growth of new phases, thereby enhancing the fracture toughness of GaTe.^11^

**Figure 3:**
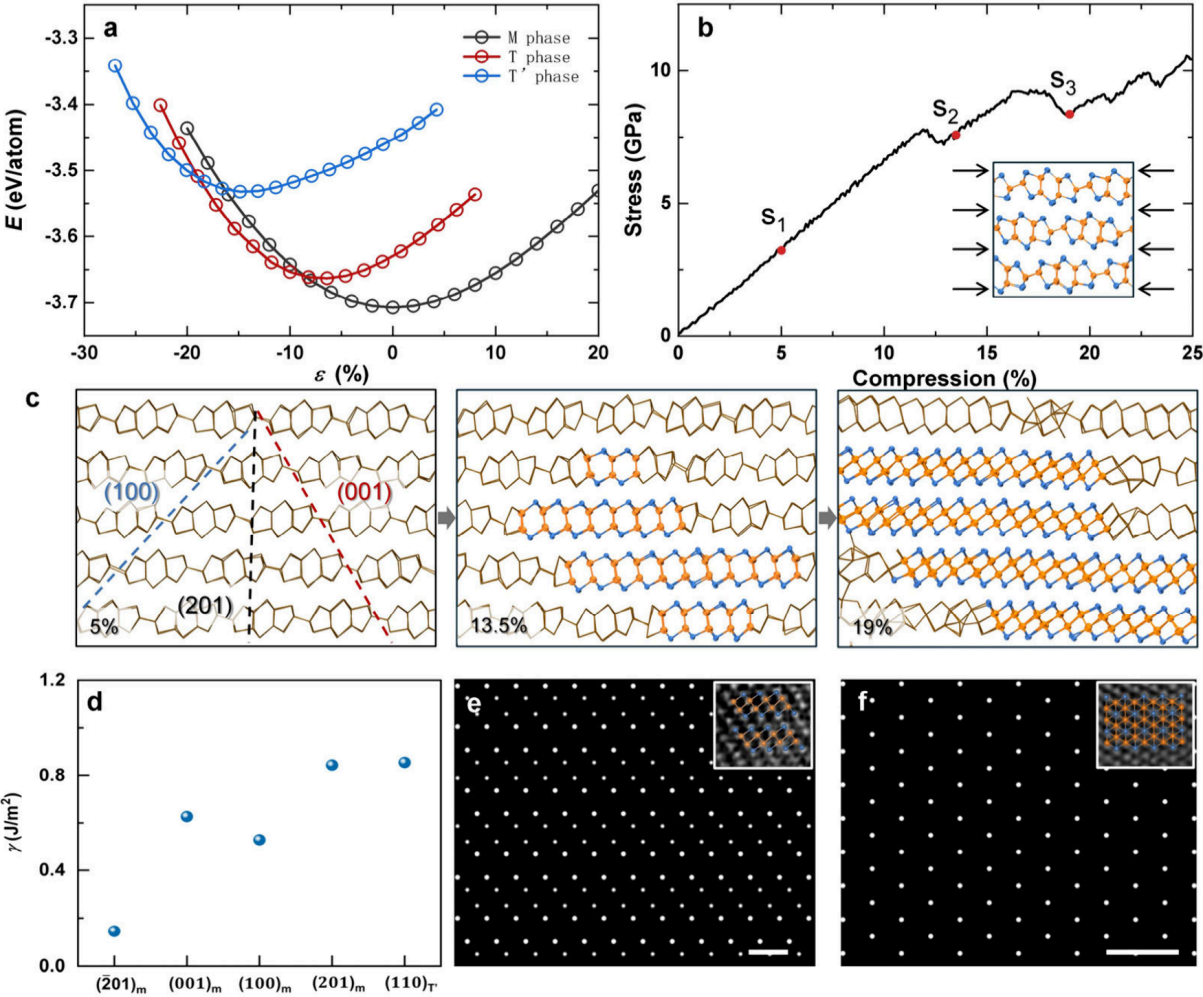
Microscopic origin of phase transformation in GaTe crystals. (a) Energy evolution of three competing phases under uniaxial compressive strain along the in-plane direction. (b) Simulated stress–strain curves of m-GaTe under lateral compression based on a tailored MLP. (c) Corresponding atomic structures under compression, with the transformed phase highlighted. (d) Calculated surface energies of m and T′ phases for GaTe crystals. (e and f) Simulated STEM images of T′-GaTe crystals along the [110] and [001] zone axes, respectively. Scale bars, 0.5 nm.

Using the neuroevolution potential framework,^56^ we further developed a tailored machine-learning potential (MLP) for GaTe crystals, enabling us to probe the kinetic pathway of phase transitions. More details about the MLP construction and validation are provided in the method section and Figure S6. Then, molecular dynamics (MD) simulations of compressive deformation were conducted on monoclinic GaTe along the in-plane [201] direction (Figure 3b). Corresponding atomic structures are presented in Figure 3c. The stresses first increase linearly with the applied strain, characterized by an elastic modulus of 66.6 GPa.^57^ As the strain exceeded 12.1%, compressive stress promotes the rotation of Ga–Ga bonds between adjacent five-membered rings, which triggers the local nucleation and growth of the T phase. Then, a second transition from the T to T′ phase occurs at a critical strain of 17.2%, mediated by the relative sliding between two intralayer Ga atomic planes. Both processes are accompanied by a pronounced stress drop, marking the onset of plastic deformation and the dissipation of strain energy. These results are in excellent agreement with our DFT calculations and experimental observations.

To rationalize the crack propagation behavior, we calculated the surface energies of the relevant fracture planes in both monoclinic and trigonal phases. As shown in panels c and d of Figure 3, the interlayer (201) plane of m-GaTe has a very low surface energy of 0.15 J/m^2^ due to weak vdW interactions, consistent with the experimentally observed brittle cleavage. In comparison, surfaces cutting across the lamellae exhibit much higher and strongly anisotropic surface energies. The (201) surface energy is 0.84 J/m^2^, while the inclined (100) and (001) planes show relatively lower values of 0.53 and 0.63 J/m^2^, respectively. Therefore, crack propagation preferentially follows the low-energy (100) and (001) planes, rationalizing the sustained crack deflection observed in experiments. Moreover, the monoclinic-to-trigonal phase transitions alter the fracture landscape at the deflection sites. Because the T′ phase possesses a higher and more homogeneous surface energy (∼0.86 J/m^2^) than the initially preferred low-energy (100) and (001) cleavage planes, the formed T′ phase increases the energetic cost of crack advance along these original paths. As a result, the crack is arrested along its initial path and subsequently redirected to the alternative crystallographic plane, i.e., from (100) to (001) or vice versa. This transformation-induced reorientation of the crack path suppresses straightforward cleavage and promotes crack deflection, thereby contributing to enhanced fracture toughness, in agreement with the experimentally observed fracture behavior. Besides, the simulated STEM images of the T′ phase along both the interlayer (110) and intralayer (001) directions closely match the experimental observations (Figure 3e,f).

With an ideal direct bandgap of ∼1.65 eV, m-GaTe holds strong potential for high-performance photodetection. Nevertheless, its practical application is hindered by the intrinsic tendency toward fragmentation and interlayer delamination, which are particularly severe in wearable electronics.^1,58^ Our findings on anisotropic fracture and phase-transformation toughening provide a viable solution to this engineering challenge. To verify this concept, flexible two-terminal photodetectors were deliberately fabricated by transferring m-GaTe crystals onto polyethylene terephthalate (PET) substrates, selected for their mechanical flexibility and optical transparency. The basal planes of m-GaTe were intentionally aligned parallel to the substrate to evaluate the out-of-plane fracture toughness during bending, and the devices were further encapsulated with a thin polystyrene (PS) layer, which serves as a protective barrier to suppress potential surface oxidation of m-GaTe while maintaining efficient light transmission (Figure 4a and Figure S9a).

**Figure 4:**
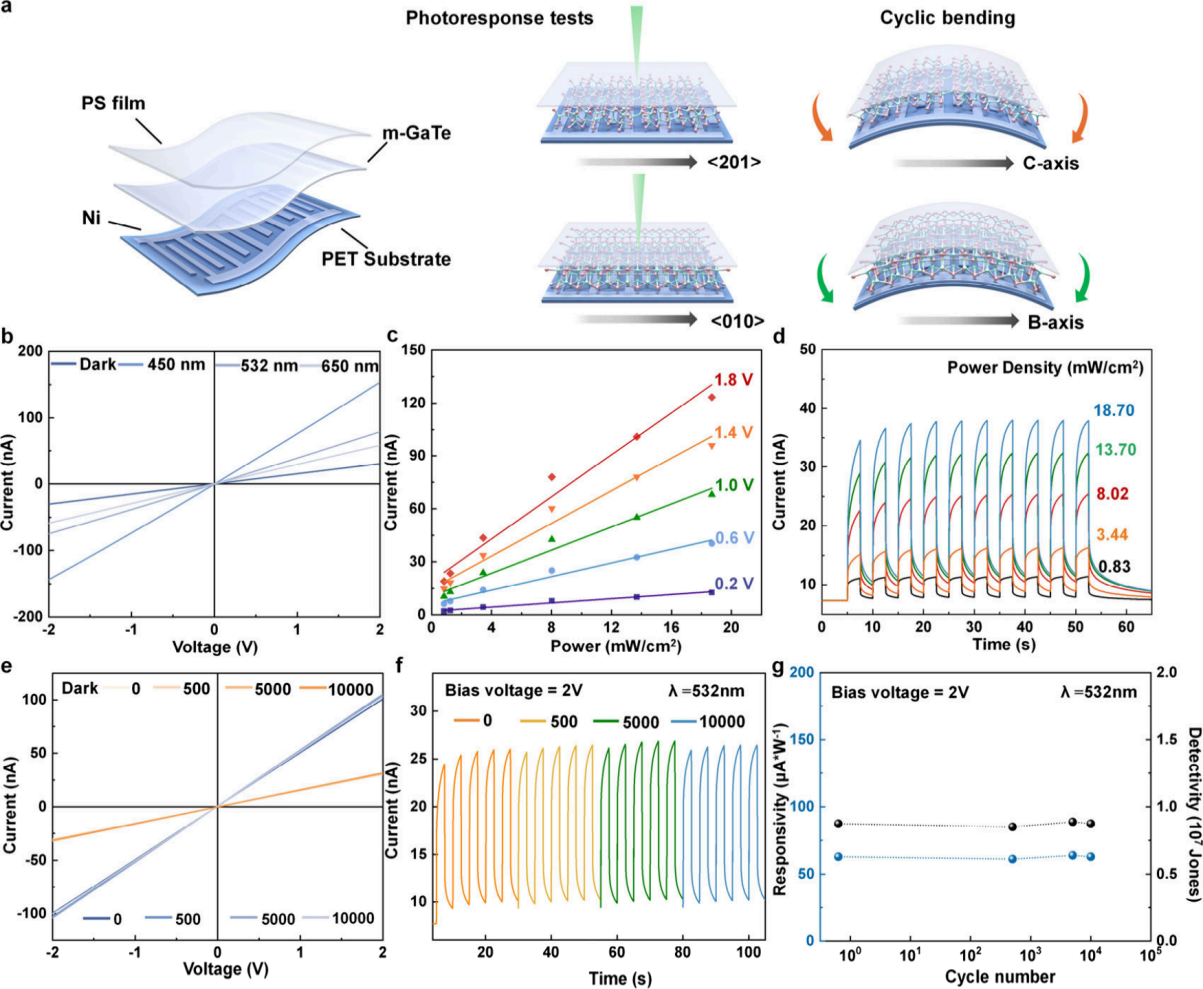
Mechanical robustness of the flexible m-GaTe photodetector. (a) Schematic of the m-GaTe photodetector and cyclic bending test setup (radius of 2.5 mm); the photoresponse was measured in the flat state after bending. (b and c) *I–V* characteristics and photocurrent of the device under varying wavelengths, optical power densities, and bias voltages (measured along the *c*-axis). (d) Photocurrent response to different light intensities at a fixed bias of 2 V (measured along the *c*-axis). (e) *I*–*V* characteristics of the device after various bending cycles (measured along the *c*-axis). (f) Evolution of the time-dependent photoresponse with an increase in the number of bending cycles (measured along the *c*-axis). (g) Responsivity (*R*) and specific detectivity (D*) of the device after successive bending cycles (measured along the *c*-axis).

The photoresponse of the device was systematically characterized along the crystallographic *c*-axis under visible light illumination at 450, 532, and 650 nm (Figure 4b). Upon illumination, the photocurrent increased monotonically with incident light intensity, indicative of efficient photocarrier generation and collection within the m-GaTe channel.^56^ Linear and symmetric current–voltage (*I*–*V*) characteristics indicate well-established, stable ohmic contacts between m-GaTe and the Ni electrode. At a fixed bias voltage of 1.8 V, the photocurrent increases from 23.5 to 123.46 nA as the light intensity is increased from 1.25 to 18.29 mW cm^–2^, respectively, demonstrating a clear intensity-dependent photoresponse (Figure 4c). In addition, the device exhibits a fast and reproducible photoresponse under repeated on/off switching cycles across different light intensities, indicating reliable photodetection performance (Figure 4d).

We next evaluated the long-term optoelectronic stability of the flexible photodetectors under repeated bending cycles applied along the crystallographic *c*-axis, corresponding to the direction of the highest fracture toughness (Figures 1f and 4a). The devices were subjected to bending with a maximum curvature of up to 0.4 mm^–1^. As shown in Figure 4e, the dark current (*I*_dark_) and photocurrent (*I*_photo_) retained their initial magnitudes even after 10 000 bending cycles, demonstrating outstanding mechanical and optoelectronic durability and indicating stable electrical transport behavior under mechanical deformation. Moreover, under a constant bias of 2 V, the operational reliability of the devices was further verified through repeated on/off illumination cycling tests during bending, in which both *I*_on_ and *I*_off_ exhibit negligible degradation and maintain their original values after 10 000 bending cycles (Figure 4f). We further calculated responsivity *R* and specific detectivity D*. The key performance parameters, *R* and D*, were evaluated before and after the bending tests, as calculated from the following equations:^59^

3 $$R=\frac{I_{\mathrm{photo}}-I_{\mathrm{dark}}}{PS}$$

4 $$D^{*}=\frac{R}{\sqrt{\frac{2qI_{\mathrm{dark}}}{S}}}$$

where *P*, *S*, and *q* denote the incident light power intensity, active area of the device, and electron charge, respectively. Under 532 nm illumination at a bias voltage of 2 V and a light intensity of 0.83 mW cm^–2^, the device exhibits a maximum responsivity of 131.94 μA W^–1^ and a specific detectivity of 1.83 × 10^7^ Jones. A decreasing trend is observed with an increase in light intensity, attributed to an increased level of recombination of photoexcited carriers at high light intensity (Figure S9b).^60^ Importantly, after 10 000 repeated bending cycles along the *c*-axis, the photodetector maintains nearly unchanged performance under 6.00 mW cm^–2^ illumination. The responsivity and detectivity stabilize at approximately 62.75 μA W^–1^ and 8.70 × 10^6^ Jones, respectively. These values are essentially identical to those obtained before bending, underscoring the intrinsic mechanical robustness and optoelectronic stability of m-GaTe along the crystallographic *c*-axis^18,25,61^ (Figure 4g). In contrast, devices bent along the *b*-axis exhibited a pronounced degradation in performance, with the *R* and D* retaining only 43.8% and 56.2%, respectively, of their initial values (corresponding to decreases in *R* from 154.52 to 67.69 μA/W and in D* from 2.51 × 10^7^ to 1.41 × 10^7^ Jones, respectively) (Figure S9e). The rapid performance decrease observed within the first 5000 cycles is attributed to the accumulation of microcracks along mechanically vulnerable crystallographic directions. These microcracks introduce physical discontinuities and localized electronic disorder in the conductive channel, which collectively hinder charge transport and suppress efficient photogenerated carrier collection, as shown in Figures S9c,d and S10.^18,25,61^

In summary, this study reveals an intrinsic toughening mechanism in the low-symmetry GaTe crystal, mediated by a compression-induced phase transformation. For the out-of-plane direction (bending along the *c*-axis), where the fracture toughness reaches a mean fracture toughness of 0.67 ± 0.09 MPa m^0.5^, the localized stress concentration at the crack tip triggers a monoclinic-to-trigonal phase transition. The newly formed T′ phase enhances fracture toughness through two synergistic effects. First, its formation facilitates stress relaxation, effectively hinders crack propagation along the (001) plane, and promotes deflection toward alternative low-surface energy planes; second, the T′ phase exhibits a higher (110) surface energy compared to those of the (100) and (001) planes of the original monoclinic phase. The combination of these factors effectively suppresses brittle cleavage, leading to remarkable ductility in the material. These insights advance our understanding of deformation mechanics in 2D van der Waals materials and open a new avenue for designing durable, flexible electronics by harnessing the intrinsic anisotropy of deformable semiconductors.

## Supplementary Material



## Data Availability

All study data are included in the article and/or Supporting Information.

## Notes

*In situ* nanomechanical testing, first-principles calculations, MLP construction and MD simulations, fabrication procedure of DCB specimens, material characterization of m-GaTe, FEM modeling of the fracture process, photodetector device fabrication, details of specimen dimensions, comparison of the mechanical robustness of different flexible photodetectors, and cyclic bending conditions for flexible m-GaTe photodetectors (PDF)
