# Supplementary material for: Activating Phase-Transition Toughening in van der Waals Semiconductor GaTe
Source: Nano Lett. 2026 Jul 7;26(29):9514–22. doi: 10.1021/acs.nanolett.6c01935 (PMC13430690; doi:10.1021/acs.nanolett.6c01935)
Supplement: Supplementary file 1 [file nl6c01935_si_001.pdf]

Supporting Information

Activating Phase-Transition Toughening in van der Waals Semiconductor GaTe

Ruihan Xu [a,#], Boxiang Gao [c,#], Danlei Zhao [d,#], Jingzhuo Zhou [a], Qi Zhu [a], Yupeng Ma [a,f], Binzhao Li [e], Yi Zhang [a], Juzheng Chen [a], Qian Zhang [a], Fanling Meng [a], Johnny C. Ho [c], Maolin Yu [b*], and Yang Lu [a,f*]*

[a] Department of Mechanical Engineering, The University of Hong Kong, Hong Kong, China.
[b] State Key Laboratory of Structural Analysis, Optimization and CAE Software for Industrial Equipment, Department of Engineering Mechanics, Dalian University of Technology, Dalian 116023, China.
[c] Department of Materials Science and Engineering, City University of Hong Kong, Kowloon, China.
[d] State Key Laboratory of High-performance Precision Manufacturing, Dalian University of Technology, Dalian, People's Republic of China.
[e] Department of Mechanical Engineering, City University of Hong Kong, Kowloon, China.
[f] Materials Innovation Institute for Life Sciences and Energy (MILES), HKU-SIRI, Shenzhen, People's Republic of China.
[#] These authors contributed equally.

** Corresponding authors. Email: johnnyho@cityu.edu.hk (J.C.H); mlyu@dlut.edu.cn (M.Y.); ylu1@hku.hk (Y.L.)*

This file includes:

Section S1. *In situ* nanomechanical testing.

Section S2. First-principles calculations.

Section S3. MLP construction and MD simulations.

Section S4. Fabrication procedure of DCB specimens.

Section S5. Material characterization of m-GaTe.

Section S6. FEM modeling of the fracture process.

Section S7. Photodetector device fabrication.

Table S1. Specimen dimensions for fracture toughness measurements.

Table S2. Comparison of the mechanical robustness of different flexible photodetectors.

Table S3. Cyclic bending conditions for flexible m-GaTe photodetectors.

Section S1. *In situ* nanomechanical testing.

In situ nanomechanical testing and microstructural characterization: *In situ* micro-fracture tests were performed using a quantitative Hysitron PI 85 PicoIndenter within an FEI Quanta 450 Field Emission SEM. *In situ* nano-fracture tests were performed on a transmission electron microscope (Thermo Scientific Talos F200X) with the PicoFemto holder (Zeptools). After the *in situ* fracture tests, the region along the crack path was examined using both high-resolution TEM and HAADF-STEM, the latter being acquired with a semi-convergence angle of about 30 mrad.

Section S2. First-principles calculations.

The first-principles calculations were carried out using the Vienna Ab-initio Simulation Package (VASP) code.[1] We employed the Perdew-Burke-Ernzerhof parametrization (PBE) of generalized gradient approximation (GGA) as the exchange-correlation potential, while the interaction between valence electrons and ionic cores was treated within the projector augmented-wave (PAW) framework.[2] A plane-wave basis set with a kinetic energy cutoff of 450 eV was employed. Brillouin-zone integrations were performed using a Γ-centered k-point grid with a reciprocal-space resolution of 0.16 $Å^{-1}$. All atomic structures were fully optimized until the residual forces on each atom were below 0.01 eV/Å and the total energy change converged to within 1×10^{-6} eV. Dispersion interactions were taken into account using the zero-damping Grimme D3 scheme.[3]

Section S3. MLP construction and MD simulations.

MLP construction and MD simulations: The machine-learning potential for GaTe was constructed using the neuroevolution potential framework implemented in the GPUMD package.[4] Based on a feedforward neural network with a single hidden layer, the site energy of an atom *i* was formulated as

$$U_i=\sum_{\mu=1}^{N_{\text{neu}}}\omega_{\mu}^{(1)}\tanh\left(\sum_{\nu=1}^{N_{\text{des}}}\omega_{\mu\nu}^{(0)}q_{\nu}^{i}-b_{\mu}^{(0)}\right)-b^{(1)} \tag{1}$$

where N_{neu} is the number of neurons, N_{des} denotes the number of descriptor vectors, and the tangent function tanh(x) is employed as the activation function. The parameters $\omega^{(0)}$ and $\omega^{(1)}$ represent the weight matrices connecting the input layer to the hidden layer and the hidden layer to the output node, respectively. $b^{(0)}$ and $b^{(1)}$ correspond to the bias terms of the hidden layer and the output node. The descriptor vectors consist of both radial and angular components. We adopted three-body and four-body angular descriptor components to increase the computational accuracy. To adequately describe long-range van der Waals interactions, relatively extended cutoff distances of 8 Å for radial terms and 4 Å for angular terms were adopted.

The training dataset was generated via an active learning strategy. Initially, a seed set consisting of 93 atomic configurations was constructed from randomly perturbed structures and snapshots extracted from *ab initio* molecular dynamics simulations. This dataset was used to train the initial NEP model and to define the basic structural descriptors. Subsequently, additional configurations were progressively collected from NEP-MD trajectories and incorporated into the training set through iterative retraining. To ensure sufficient configurational diversity during dataset enrichment, a farthest-point sampling scheme was employed with a descriptor-distance cutoff of 0.05. The explored trajectories spanned a wide range of structural and thermodynamic conditions (0-800 K), including multiple phases, edge structures, crack configurations, and mechanically deformed states. This active learning loop was repeated until the model exhibited consistently low prediction errors on newly acquired configurations, indicating convergence of the training dataset. The resulting dataset comprises 2,881 configurations, among which 90% were used for training and 10% for evaluation. The trained model achieves root-mean-square errors below 1.4 meV/atom for energies and 127.2 meV/Å for forces on both the training and test sets. MD simulations were performed using the large-scale atomic/molecular massively parallel simulator (LAMMPS) package.[5] The simulation boxes had approximate dimensions of

$120{\times}8.3{\times}110$ Å^3, and a time step of 1 fs was employed. Before loading, all systems were subjected to energy minimization followed by dynamic equilibration at 300 K for 100 ps. Then, compression was applied to the samples by changing their lattice constant along the in-plane direction at a specific rate of 1×10^8 /s. A canonical (NVT) ensemble was used to maintain fixed cell shape throughout the simulation.

Section S4. Fabrication procedure of DCB specimens

For DCB testing,[6-8] a lamella (35 μm × 7 μm × 2.5 μm) was lifted from the bulk m-GaTe crystal. FIB preparation involved initial rough milling (30 kV, 2.5 nA) to trench and isolate three specimens, followed by progressive thinning of their central regions to electron transparency at 80, 40, and 24 pA. A 1 μm-long pre-crack notch was introduced at the center of the thinned region using a 7 pA. A final low-voltage polish at 5 kV / 10 pA was applied to remove amorphous damage. The DCB specimen features two thick arms (~2.5 μm) and a central electron-transparent region (100 ± 20 nm), with the notch serving as a stress concentrator to initiate crack propagation (Figure S2).

Section S5. Material characterization of m-GaTe.

Crystal structure and morphology were characterized using X-ray diffraction (XRD; Bruker D8 Advance, Cu Kα radiation), scanning electron microscopy (SEM; FEI Quanta 450 FEG), and high-resolution transmission electron microscopy (HRTEM; Thermo Scientific Talos F200X). Additional microstructural analysis was performed using high-angle annular dark-field scanning transmission electron microscopy (HAADF-STEM; Spectra Ultra STEM). Raman spectroscopy, photoluminescence (PL) spectroscopy, and spectral mapping were conducted on a confocal microscopy spectrometer (WITec Alpha 300R) with a 532 nm laser source operating at 2 mW power. Polarization-dependent measurements were performed by rotating the incident laser direction in a parallel configuration. The simulations of STEM-HAADF images were conducted with the Dr. Probe software,[9] with the accelerating voltage (300 kV), convergence semi-angle (30 mrad), and collection angle (52–200 mrad) set to mirror the experimental conditions.

X-ray diffraction (XRD) pattern reveals the monoclinic phase of specimens before

micro-fracture testing, with no evidence of secondary phase formation (Figure S3a).[10] Figure S3b demonstrates the Raman spectrum of m-GaTe with five representative Ag modes between 100 and 300 cm^{-1} (at 108, 114, 207, 268, and 280 cm^{-1}). The absence of peaks at 132 and 146 cm^{-1} indicates no detectable oxygen contamination, consistent with the known spectral signature of GaTe[11]. High-resolution STEM-HAADF images of m-GaTe captured along [010] direction, and the corresponding selected-area electron diffraction (SAED) pattern exhibit exclusively single-crystal diffraction spots. These observations confirm the excellent crystalline quality and definitively exclude FIB-related crystalline damage and amorphization (Figures S3c and S3d).

Section S6. FEM modeling of the fracture process

The cohesive zone model (CZM) was employed to analyse the strain field distribution during crack propagation in the off-plane direction. The CZM focuses on the fracture process zone ahead of the crack tip, governed by a traction–separation law.[12] In the finite element model, this law is implemented through cohesive elements. inserted along the potential crack path. Two key parameters define the cohesive behavior: the maximum strength T_{max}, and the failure displacement δ_f. The parametric analysis was conducted by varying T_{max} and δ_f as performed to identify the set of parameters that best reproduces the experimental results.[13] The crack opening displacement near the tip can be related to the material's fracture toughness using Irwin's equation.

$$\mu(x)=\frac{K_{IC}}{E}\sqrt{\frac{8x}{\pi}} \quad (1)$$

Where 2μ is the crack opening at a distance x behind the crack tip, K_{IC} is the mode I fracture toughness, and E is the Young's modulus. Figure S5a compares the experimental and simulation results using a maximum traction T_{max} = 500 MPa and a failure displacement. δ_f= 145 nm. The scatter points in Figure S5a represent the discrete local crack opening displacements, which were extracted from sequential frames of the FEM simulations during stable crack propagation. By fitting these discrete

data points to Irwin's equation, we determined the simulated fracture toughness. Notably, both the simulated crack-opening profiles and the extracted fracture toughness demonstrate excellent agreement with the experimental measurements (Figure S5a), thereby robustly validating our continuum theoretical model.

Furthermore, Figure S5b provides an intuitive visualization of the fracture process, displaying four sequential stages of strain evolution during the simulated DCB test. As the wedge-shaped diamond indenter (half-angle ~55 °) advances downward at a constant speed of ~4 nm/s to progressively separate the crack surfaces, the contour maps dynamically capture the strain distribution. Here, the color gradient from blue to red indicates the local transition from tensile to compressive strains. Specifically, the first sub-figure illustrates the critical state just prior to the initial crack deflection, where localized compressive strains reaching ~7.0% emerge at the deflection sites. It is worth noting that, compounded by the intrinsic structural singularity at the crack tip, the actual near-tip strains in the material are expected to be even more intensely amplified than those predicted by these continuum simulation results.

Section S7. Photodetector device fabrication

For the fabrication of p-type flexible GaTe-based devices, 50 nm-thick nickel (Ni) films were deposited as contact electrodes via thermal evaporation through a pre-patterned mask on a PET substrate, achieving a defined channel length of 100 μm. A custom-prepared polystyrene (PS) solution (90 mg/mL) was spin-coated at 3000 rpm for 1 min onto a SiO_2/Si substrate bearing an exfoliated GaTe flake, and subsequently annealed at 80 °C for 1 min. The substrate was then immersed in deionized water to promote the detachment of the PS film, which was thereafter carefully retrieved, microscopically aligned, and transferred onto the pre-deposited electrodes to realize bottom-contact device architectures.

The photoresponse of the devices was characterized in a two-terminal configuration using a Lake Shore CRX-VF cryogenic probe station coupled with a Keysight B1500A semiconductor parameter analyzer. All electrical measurements were performed under both dark and laser-illuminated conditions, with the incident

optical power precisely calibrated by a Thorlabs PM400 power meter. Cyclical bending tests were conducted on a micro-tester (MINi-MTS500, Beijing Qiyue Technology Co., Ltd.) under displacement control. A 125 μm thick polyethylene terephthalate (PET) substrate was clamped at its two edges between a stationary and a movable fixture, enabling precise control of the bending radius down to 2.5 mm. The photoresponse properties were systematically re-evaluated after predetermined numbers of bending cycles. The photoresponse properties were systematically measured along b and c directions (Figures 4 and S7). [14]

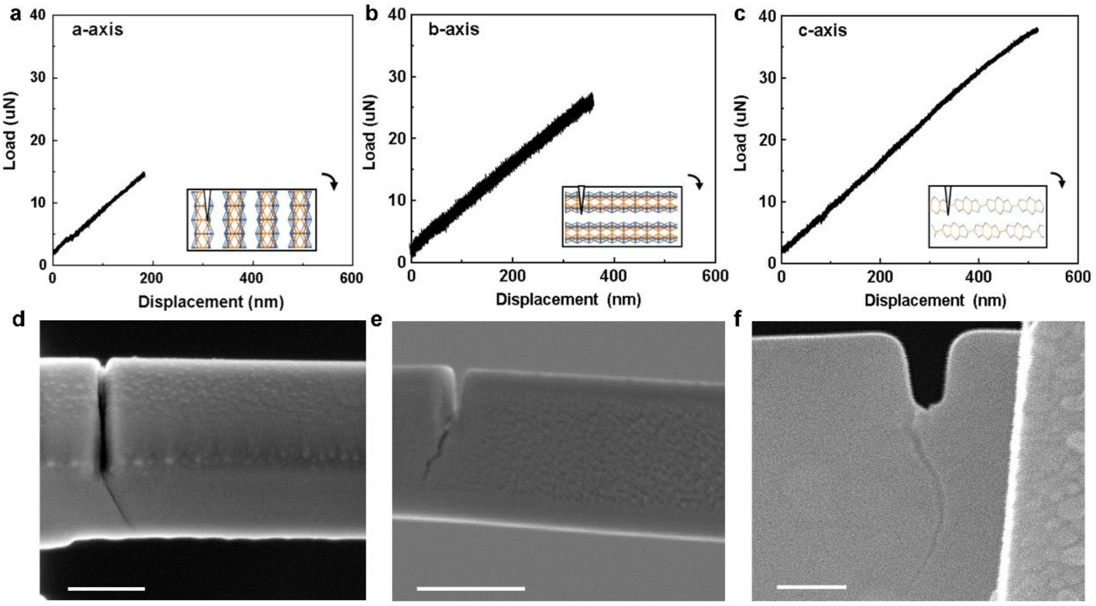

Figure S1. Fracture anisotropy in m-GaTe crystals. (a-c) show the load-displacement curves along a-, b-, and c-axes. (d-f) present the corresponding fracture morphologies. Scale bars, 1 μm, 1 μm, and 0.3 μm, respectively.

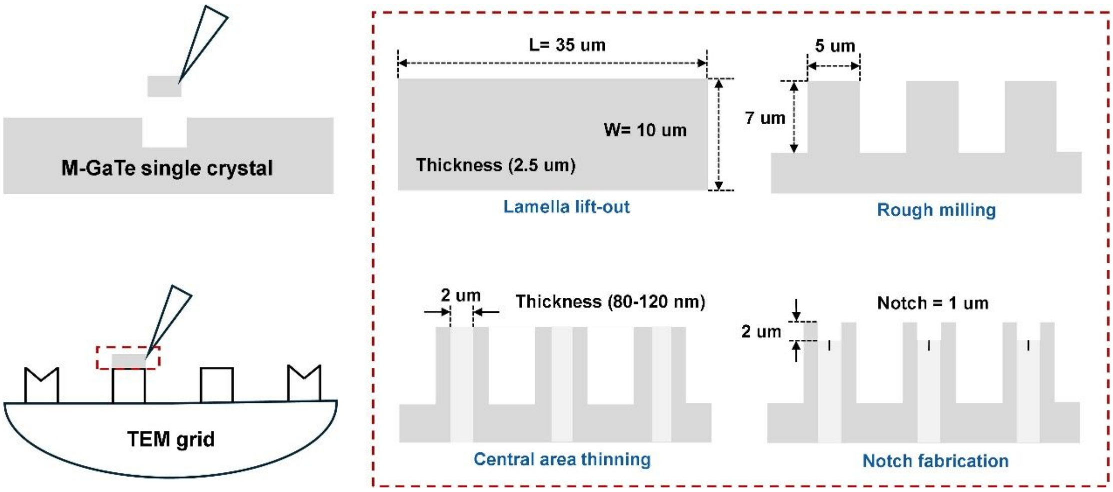

Figure S2. Fabrication processes of the m-GaTe DCB setup.

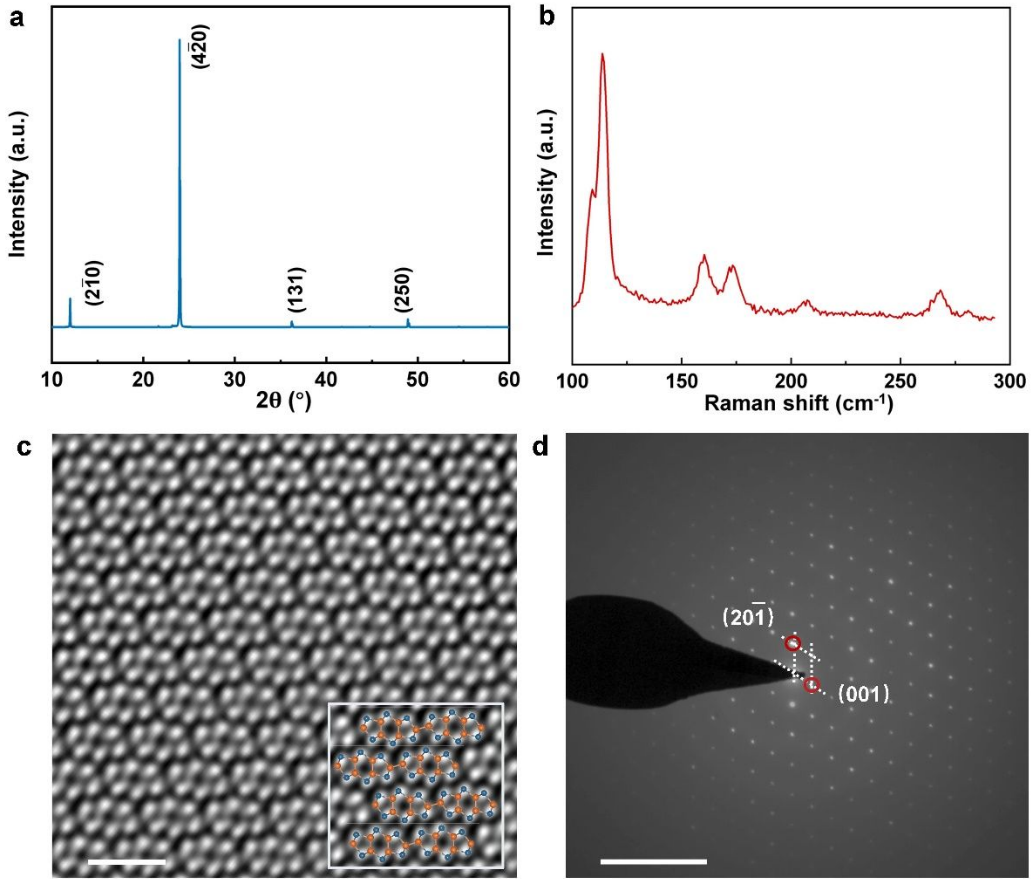

Figure S3. Material characterization of bulk m-GaTe before fracture testing. (a) XRD patterns of the m-GaTe samples prepared for in situ fracture experiments. (b) Raman spectrum of m-GaTe obtained using a 532 nm laser. (c) High-resolution STEM-HAADF image of m-GaTe along the [010] direction. Scale bar, 2 nm. (d) The corresponding SAED pattern. Scale bar, 5 nm^{-1}.

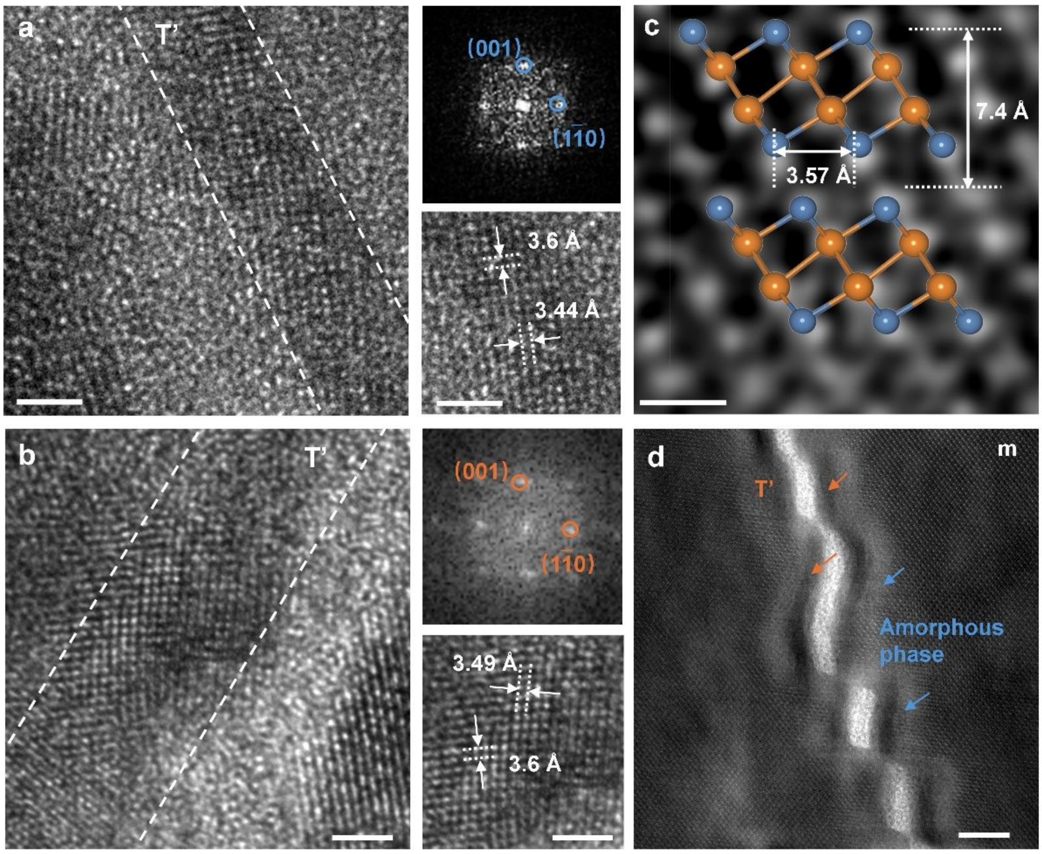

Figure S4. Phase evolution along the crack. (a, b) HRTEM image and the corresponding Fast Fourier Transform (FFT) pattern ([110] zone axis) of the trigonal T′ phase at the crack deflection. Scale bars, 2 nm. (c) Atomic structure of the T′ phase. The labeled spacing of 3.57 Å corresponds to the d-spacing of the (110) planes, which yields a calculated lattice constant of a = 4.10 Å. Scale bar, 0.4 nm. (d) Distribution of T′ and amorphous phases along the crack path. Scale bar, 2 nm.

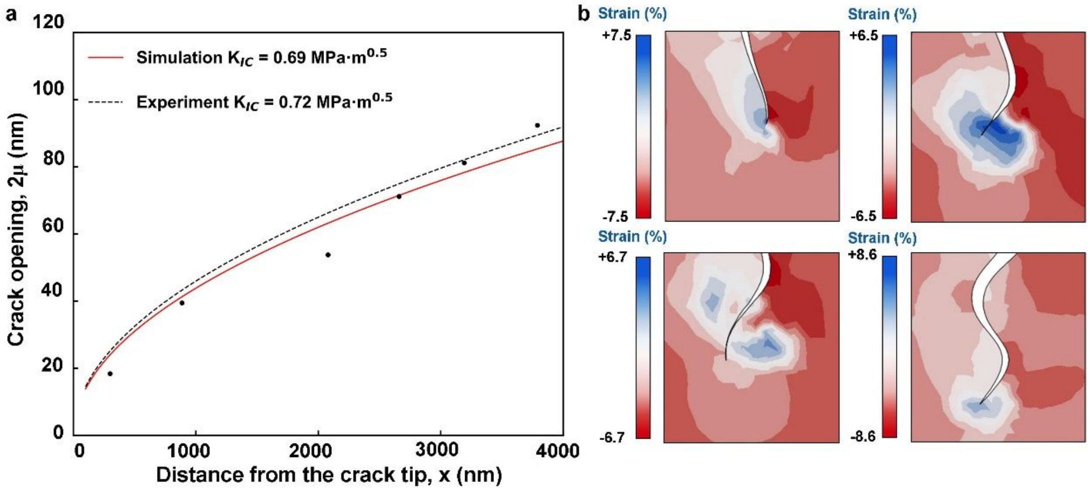

Figure S5. The finite element method (FEM) simulation of DCB fracture testing. (a) Comparison of the simulated versus experimental fracture toughness. (b) The corresponding strain field evolution during the crack propagation stages.

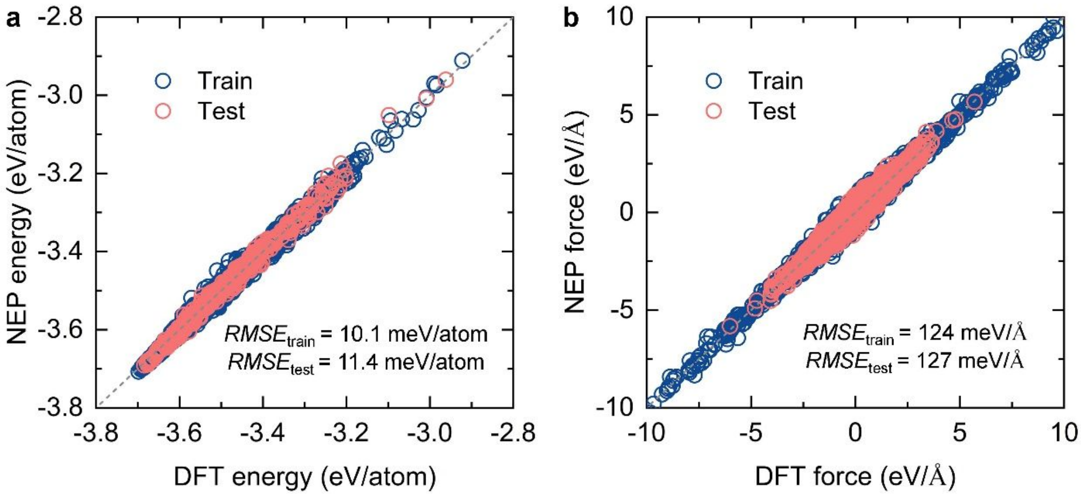

Figure S6. Construction and assessment of the NEP model for the GaTe system. (a) Energy and (b) force parity plots for training and testing datasets.

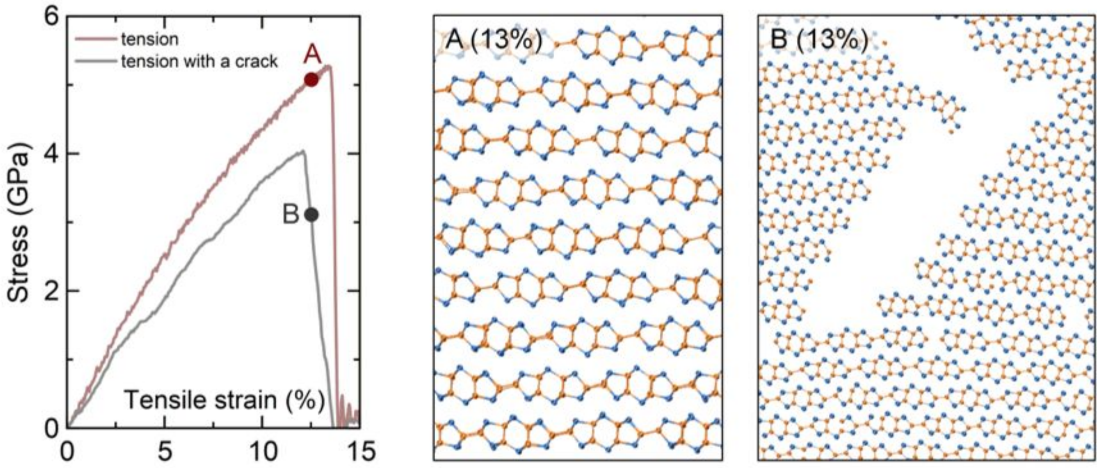

Figure S7. Tensile stress-strain responses of m-GaTe crystals with and without a pre-crack. Detailed atomic structures at a strain of 13% are shown in the middle and right panels.

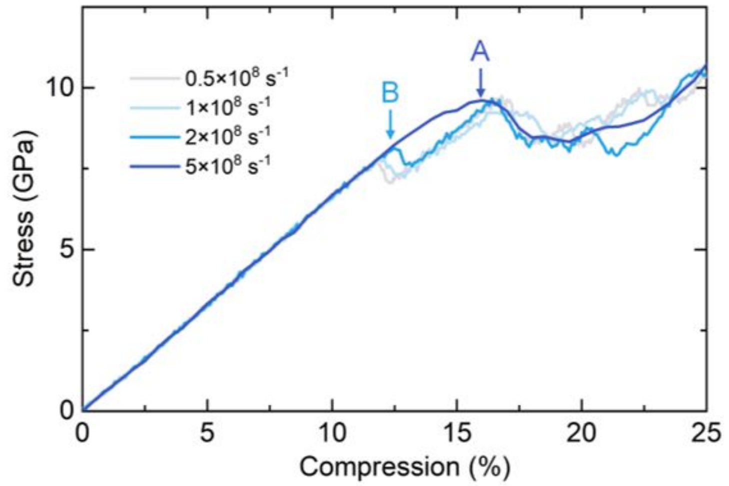

Figure S8. Compressive stress-strain responses of m-GaTe crystals at different strain rates.

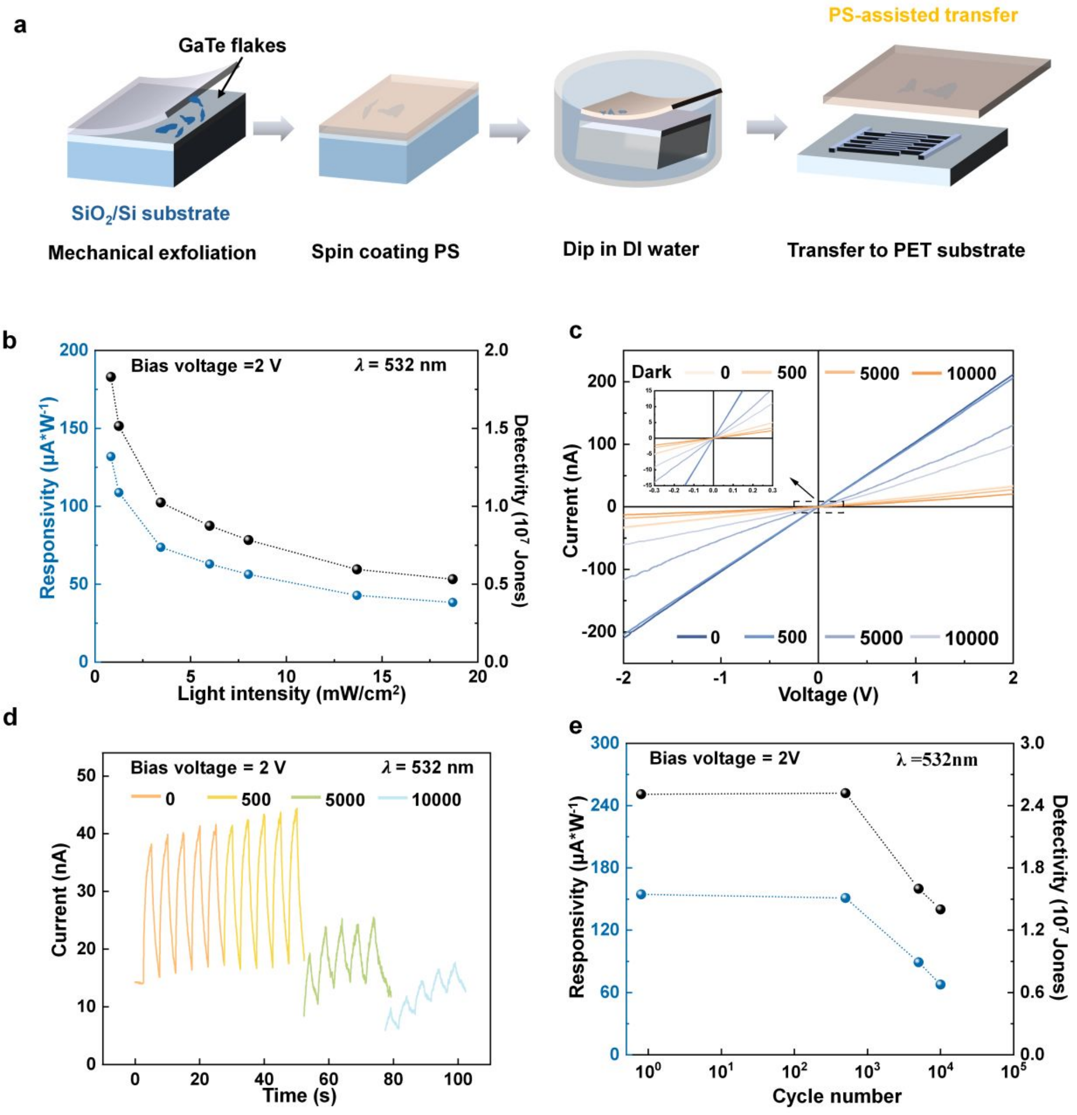

Figure S9. Performance and bending durability of flexible m-GaTe photodetectors. (a) Schematic illustration of the device fabrication process. (b) Responsivity (R) and specific detectivity (D*), measured along the c-axis, as functions of incident power density. (c) Degradation of I-V characteristics after multiple bending cycles (measured and bending along the b-axis). (d) Evolution of the temporal photoresponse with increasing bending cycles (measured and bending along the b-axis). (e) Responsivity (R) and specific detectivity (D*) of the device after successive bending cycles (measured and bending along the b-axis).

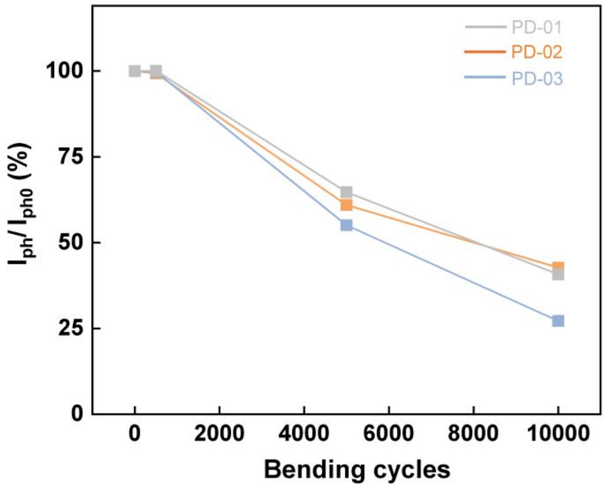

Figure S10. Photocurrent (I_{ph}) change of flexible m-GaTe photodectors bent along the b-axis after cyclic bendings.

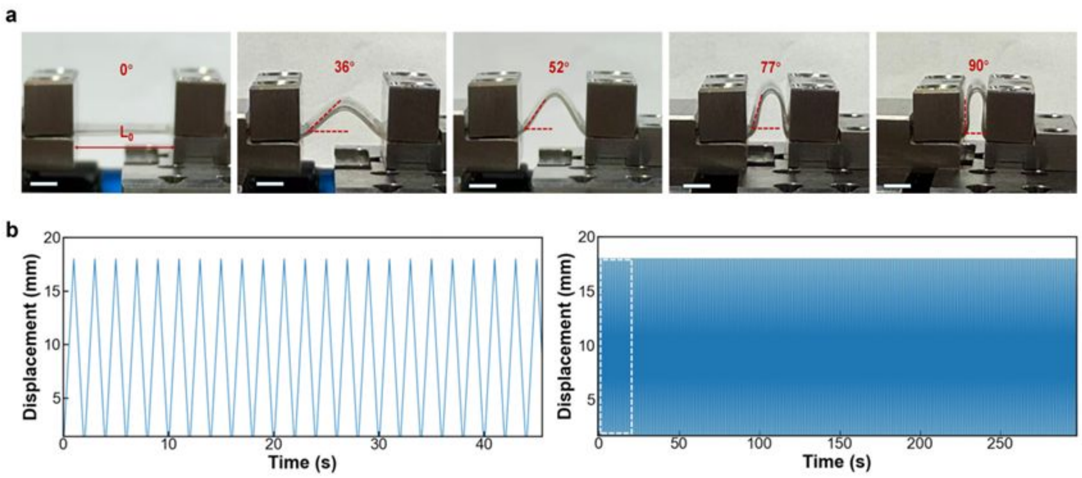

Figure S11. Experimental setup for the cyclic bending tests. (a) m-GaTe flexible photodetector at various bending angles. Scale bars, 6.58mm. (b) Displacement-time

curves of the cyclic loading.

Table S1. Specimen dimensions for fracture toughness measurements.

Crystal orientation			L (μm)	W (μm)	B (μm)	a (μm)
Out-of-plane	c axis	c_1	4.78	1.08	1.19	0.29
		c_2	5.61	1.18	1.24	0.30
		c_3	4.83	1.16	0.98	0.21
		c_4	4.64	1.40	1.38	0.24
	b axis	b_1	3.91	0.95	1.06	0.21
		b_2	4.59	1.13	1.06	0.35
		b_3	4.82	0.97	0.99	0.20
		b_4	4.68	1.25	0.99	0.25
In-plane	a axis	a_1	4.93	1.05	0.90	0.13
		a_2	5.38	1.30	1.09	0.32
		a_3	3.95	0.65	0.537	0.14

Table S2. Comparison of the mechanical robustness of different flexible photodetectors.

Materials		Bending Angle	Bending cycles	Photocurrent changes (I_{ph}/I_{ph0})	References
m-GaTe	c-axis	90°	10000	99.47%	This work
	b-axis	90°	10000	36.89%	
SnSe		60°	200	89.3%	15
$CdS_{0.14}Se_{0.86}$		-	50	28.6%	16
Organolead triiodide perovskite ($CH_3NH_3PbI_3$)		80°	200	89.29%	17
PbI_2		60°	100	71%	18
$CsPbBr_3$		60°	1500	85%	19

Table S3. Cyclic bending conditions for flexible m-GaTe photodetectors.

Bending conditions

Bending Mode	Uniaxial Bending
Loading Mode	Displacement control, constant linear loading rate
Bending Frequency (cycles/min)	30
L_0 (Initial Spacing, mm)	23
L (Minimum Spacing, mm)	5
Bending Angle (°)	90
Strain (%)	2.5
Bending Radium (mm)	2.5
Bending Axis Orientations	b-, and c-axes
Numbers of Bending Cycles	0,500,5000 and 10000

References

(1) Kresse, G.; Furthmüller, J. Efficiency of Ab-Initio Total Energy Calculations for Metals and Semiconductors Using a Plane-Wave Basis Set. *Comput. Mater. Sci.* 1996, 6, 15–50.

(2) Perdew, J. P.; Burke, K.; Ernzerhof, M. Generalized Gradient Approximation Made Simple. *Phys. Rev. Lett.* 1996, 77, 3865–3868.

(3) Grimme, S.; Antony, J.; Ehrlich, S.; Krieg, H. A Consistent and Accurate *Ab Initio* Parametrization of Density Functional Dispersion Correction (DFT-D) for the 94 Elements H-Pu. *J. Chem. Phys.* 2010, 132, 154104.

(4) Fan, Z.; Wang, Y.; Ying, P.; Song, K.; Wang, J.; Wang, Y.; Zeng, Z.; Xu, K.; Lindgren, E.; Rahm, J. M.; Gabourie, A. J.; Liu, J.; Dong, H.; Wu, J.; Chen, Y.; Zhong, Z.; Sun, J.; Erhart, P.; Su, Y.; Ala-Nissila, T. GPUMD: A Package for Constructing Accurate Machine-Learned Potentials and Performing Highly Efficient Atomistic Simulations. *J. Chem. Phys.* 2022, 157, 114801.

(5) S. Plimpton, Fast parallel algorithms for short-range molecular dynamics. *J. Comput. Phys.* **117**, 1–19 (1995)

(6) Sernicola, G.; Giovannini, T.; Patel, P.; Kermode, J. R.; Balint, D. S.; Britton, T. B.; Giuliani, F. In Situ Stable Crack Growth at the Micron Scale. *Nat. Commun.* 2017, 8, 108.

(7) Zhu, Q.; Li, Z.; Wei, S.; Zhao, Y.; Ramamurty, U.; Wang, J.; Gao, H. A Deformation Twin Mediated Sliding-Opening Zig-Zag Fracture Mechanism in Multi-Principal Element Alloys. *Acta Mater.* 2024, 275, 120073.

(8) Gavalda-Diaz, O.; Emmanuel, M.; Berenov, A.; Marquardt, K.; Saiz, E.; Giuliani, F. Observing the Crack Tip Behavior at the Nanoscale during Fracture of Ceramics. *Proc. Natl. Acad. Sci.* 2024, 121, e2408292121.

(9) Barthel, J. Dr. Probe: A Software for High-Resolution STEM Image Simulation. *Ultramicroscopy* 2018, 193, 1–11.

(10) Tan, J.; Zhang, H.; Wang, X.; Wang, Y.; Wang, J.-J.; Zhang, H.; Ma, E.; Zhang,

W. Deformable Monoclinic Gallium Telluride with High In-Plane Structural Anisotropy. *Mater. Today* 2024, 80, 250–261.

(11) Hoang, N. T.; Lee, J.-H.; Vu, T. H.; Cho, S.; Seong, M.-J. Thickness-Dependent in-Plane Anisotropy of GaTe Phonons. *Sci. Rep.* 2021, 11, 21202.

(12) Heidari-Rarani, M.; Sayedain, M. Finite Element Modeling Strategies for 2D and 3D Delamination Propagation in Composite DCB Specimens Using VCCT, CZM and XFEM Approaches. *Theor. Appl. Fract. Mech.* 2019, 103, 102246.

(13) Hirakata, H.; Akiyoshi, M.; Masuda, R.; Shimada, T. High Fracture Toughness in van Der Waals-Layered MoTe2: Disappearance of Stress Singularity. *Eng. Fract. Mech.* 2023, 277, 108974.

(14) Wang, H.; Chen, M.-L.; Zhu, M.; Wang, Y.; Dong, B.; Sun, X.; Zhang, X.; Cao, S.; Li, X.; Huang, J.; Zhang, L.; Liu, W.; Sun, D.; Ye, Y.; Song, K.; Wang, J.; Han, Y.; Yang, T.; Guo, H.; Qin, C.; Xiao, L.; Zhang, J.; Chen, J.; Han, Z.; Zhang, Z. Gate Tunable Giant Anisotropic Resistance in Ultra-Thin GaTe. *Nat. Commun.* 2019, 10, 2302.

(15) Xu, H.; Hao, L.; Liu, H.; Dong, S.; Wu, Y.; Liu, Y.; Cao, B.; Wang, Z.; Ling, C.; Li, S.; Xu, Z.; Xue, Q.; Yan, K. Flexible SnSe Photodetectors with Ultrabroad Spectral Response up to 10.6 Mm Enabled by Photobolometric Effect. *ACS Appl. Mater. Interfaces* 2020, 12, 35250–35258.

(16) Xia, J.; Zhao, Y.-X.; Wang, L.; Li, X.-Z.; Gu, Y.-Y.; Cheng, H.-Q.; Meng, X.-M. Van Der Waals Epitaxial Two-Dimensional CdS_x $Se_{(1-x)}$ Semiconductor Alloys with Tunable-Composition and Application to Flexible Optoelectronics. *Nanoscale* 2017, 9, 13786–13793.

(17) Deng, H.; Yang, X.; Dong, D.; Li, B.; Yang, D.; Yuan, S.; Qiao, K.; Cheng, Y.-B.; Tang, J.; Song, H. Flexible and Semitransparent Organolead Triiodide Perovskite Network Photodetector Arrays with High Stability. *Nano Lett.* 2015, 15, 7963–7969.

(18) Zhong, M.; Huang, L.; Deng, H.-X.; Wang, X.; Li, B.; Wei, Z.; Li, J. Flexible Photodetectors Based on Phase Dependent PbI_2 Single Crystals. *J. Mater. Chem.*

C 2016, 4, 6492–6499.

(19) Deng, W.; Huang, H.; Jin, H.; Li, W.; Chu, X.; Xiong, D.; Yan, W.; Chun, F.; Xie, M.; Luo, C.; Jin, L.; Liu, C.; Zhang, H.; Deng, W.; Yang, W. All-Sprayed-Processable, Large-Area, and Flexible Perovskite/MXene-Based Photodetector Arrays for Photocommunication. *Adv. Opt. Mater.* 2019, 7, 1801521.
